# Evaluation of Two New Commercial Tests for the Diagnosis of Acute Dengue Virus Infection Using NS1 Antigen Detection in Human Serum

**DOI:** 10.1371/journal.pntd.0000280

**Published:** 2008-08-20

**Authors:** Philippe Dussart, Laure Petit, Bhety Labeau, Laetitia Bremand, Alexandre Leduc, David Moua, Séverine Matheus, Laurence Baril

**Affiliations:** 1 Centre National de Référence des Arbovirus et Virus Influenza, Région Antilles-Guyane, Institut Pasteur de la Guyane, Cayenne, French Guiana; 2 Unité d'Epidémiologie des Maladies Emergentes, Institut Pasteur, Paris, France; 3 Unité d'Epidémiologie des Maladies Infectieuses, Institut Pasteur de Dakar, Dakar, Sénégal; University of California Berkeley, United States of America

## Abstract

**Background:**

We compared the performance of two new commercial tests for the detection of dengue NS1 protein during the clinical phase of dengue virus (DENV) infection—an immunochromatographic test allowing rapid detection of the NS1 antigen, Dengue NS1 Ag STRIP (Bio-Rad Laboratories - Marnes La Coquette, France), and a two-step sandwich-format microplate enzyme-linked immunosorbent assay (ELISA), pan-E Dengue Early ELISA (Panbio - Brisbane, Australia)—with a one-step sandwich-format microplate ELISA, the Platelia Dengue NS1 Ag test (Bio-Rad).

**Methods:**

We tested 272 serum samples from patients with dengue disease. Of these, 222 were from patients with acute infection of one of the four dengue serotypes, detected by RT-PCR and/or virus isolation. Forty-eight acute-phase serum samples from patients not infected with dengue virus were also included.

**Results:**

The sensitivity of the Platelia Dengue NS1 Ag test on acute serum samples (n = 222) was 87.4% (95% confidence interval: 82.3% to 91.5%); that of Dengue NS1 Ag STRIP was 81.5% (95% CI: 75.8% to 86.4%) after 15 minutes and 82.4% (95% CI: 76.8% to 87.2%) after 30 minutes. Both tests had a specificity of 100% (97.5% CI, one-sided test: 92.6% to 100.0%). The pan-E Dengue Early ELISA had a sensitivity of 60.4% (95% CI: 53.4% to 66.8%) and a specificity of 97.9% (95% CI: 88.9% to 99.9%).

**Conclusion:**

Our findings support the use of diagnostic tools based on the NS1 antigen detection for the diagnosis of acute DENV infection. The immunochromatographic test, Dengue NS1 Ag STRIP—the first rapid diagnostic test for DENV infection—was highly sensitive and specific, and would therefore be a suitable first-line test in the field. The pan-E Dengue Early ELISA was less sensitive than the Platelia test; this two-step ELISA should be combined with DENV IgM antibody detection for the diagnosis of DENV infection.

## Author Summary

Dengue is a viral disease transmitted by mosquitoes that is endemic in more than 100 countries in tropical areas, threatening over 2.5 billion people. It causes a wide range of symptoms and has severe forms. In reference laboratories, dengue disease is confirmed by virus isolation or genome detection during the acute phase, and by serological methods during the early convalescent phase. The viral NS1 protein circulates in the sera of infected patients throughout the clinical phase of the disease. Novel diagnostic tests based on NS1 detection have been recently developed and marketed. We compared the performance of two tests for detecting dengue NS1 protein during the clinical phase of dengue infection (an immunochromatographic test (ICT) from Bio-Rad allowing rapid detection of the NS1 antigen and a two-step sandwich-format ELISA from Panbio) with the one-step sandwich-format microplate ELISA (Bio-Rad). The ICT test performed better than the ELISA test from Panbio. This study confirms that diagnostic tests based on NS1 could be used in routine clinical practice in poorly equipped laboratories and that dengue diagnosis could therefore be confirmed without the need for testing in reference laboratories. This represents a crucial step towards the control of dengue disease in the human population.

## Introduction

Dengue virus (DENV) is a mosquito-borne virus (family *Flaviviridae*, genus *Flavivirus*). Four viral serotypes of DENV (DENV-1 to DENV-4) cause disease in humans. Symptoms range from mild fever, the most common form, to potentially fatal dengue hemorrhagic fever (DHF) and dengue shock syndrome (DSS) or encephalitis and hepatitis. The disease is now endemic in more than 100 countries in tropical areas, threatening over 2.5 billion people. It currently affects around 100 million people every year [1]. Among affected individuals, 500,000 have DHF and around 25,000, mostly children, die. According to World Health Organization estimates, the incidence of dengue disease has increased by a factor of 30 over the past 50 years [2]. Many of these epidemiological changes can be attributed to the increase and spread of the vector *Stegomyia aegypti* (formerly *Aedes aegypti*), an urban mosquito, together with population growth, urbanization and increases in human travel [3].

Flaviviruses are enveloped, single-stranded, positive-sense RNA viruses. The genomic RNA is about 11 kb long and contains 10 genes encoding three structural proteins (capsid [C], envelope [E], and membrane [M]), and seven nonstructural proteins (NS1, NS2a, NS2b, NS3, NS4a, NS4b, and NS5) [4]. The polycistronic coding region is flanked by non-coding regions at its 5′ and 3′ ends. The nonstructural protein, NS1, is a highly conserved glycoprotein, but its biological activity has not been established. During *in vitro* infection, the flavivirus NS1 protein is expressed as an intracellular membrane-associated form essential for viral replication [5],[6] or as a cell surface-associated form that may be involved in signal transduction [7]. In solution, secreted NS1 protein behaves as a hexamer; it circulates and accumulates in the sera of dengue virus-infected patients throughout the clinical phase of the disease [8]–[10]. A recent study demonstrated that soluble NS1 protein binds to endothelial cells and, following recognition by anti-NS1 antibodies, could contribute to plasma leakage during severe dengue virus infection [11].

The detection of secreted NS1 protein represents a new approach to the diagnosis of acute dengue infection. A recently developed commercially available diagnostic test based on dengue NS1 antigen-capture ELISA (Platelia Dengue NS1 Ag test, Bio-Rad Laboratories, Marnes la Coquette, France), was investigated in two studies (one in South America and the other in Southeast Asia); the test had an overall sensitivity of 88.7% and 93.4% in the two studies, with 100% specificity [12],[13].

We evaluated and compared the performance of the Platelia Dengue NS1 Ag test from Bio-Rad Laboratories with two new commercial tests for the detection of dengue virus NS1 antigen (Ag) in patients with clinically diagnosed DENV infection: Dengue NS1 Ag STRIP, an immunochromatographic assay developed by Bio-Rad Laboratories, and pan-E Dengue Early ELISA, an enzyme-linked immunosorbent assay developed by Panbio (Brisbane, Australia).

## Materials and Methods

### Clinical samples

We used a panel of human serum samples from the collection of the *Centre National de Référence des Arbovirus et virus Influenza* (the French National Reference Center (NRC) for Arboviruses and *Influenza* viruses), *Région Antilles-Guyane*, based at the *Institut Pasteur de la Guyane*. Sera were collected from patients previously diagnosed with Dengue infection. No clinical investigation was conducted and no personal identifiers were included; therefore, no IRB approval was sought for this study. The laboratory as NRC conducted the collection of serum samples with approval from the French Ministry of Health. The database used by the NRC is registered with the *Commission Nationale de l'Informatique et des Libertés* (CNIL). This database provided clinical information about the age and sex of each patient, the date of serum collection and the day on which symptoms occurred (onset of fever taken as day 0, *i.e.* first 24 hours). Clinical data and serum samples were collected from patients presenting a dengue-like illness in the context of the dengue surveillance activity of the NRC.

### Dengue infection diagnosis

DENV was isolated by cell culture and the DENV genome was detected by RT-PCR, as previously described, in acute-phase serum samples [14],[15]. Briefly, for virus isolation, acute-phase serum samples from febrile patients were diluted 10-fold in Leibowitz medium containing 3% fetal calf serum, and dilutions were injected into subconfluent AP 61 cell cultures, as previously described. After 7 days of culture, cells were harvested, and dengue viruses were identified according to serotype by an indirect immunofluorescence assay (IFA) with monoclonal antibodies specific for DENV-1, -2, -3, and -4 viruses (provided by CDC, Fort Collins, CO, USA). DENV was isolated from 104 samples, and DENV RNA was detected in an additional 82 samples by RT-PCR. A third set of 36 samples was positive for both virus isolation and RT-PCR. Based on the epidemiological situation for dengue in French Guiana, we considered patients with positive detection of the genome associated with dengue-like syndrome to be confirmed cases of dengue infection.

Acute- and early convalescence-phase serum samples were tested for IgM antibodies against DENV, using the MAC-ELISA test, as previously described [16]. All sera were tested for the presence of DENV IgG antibody using a test adapted from a previously described indirect ELISA [17]. Briefly, the wells of flat-bottomed microplates (Polysorp, Nunc) were coated with 100 µl of dengue antigen diluted 1∶2,000 in phosphate-buffered saline (PBS). Normal mouse brain antigen was diluted in an identical manner and used to coat other adjacent wells. After overnight incubation at 4°C, the microplates were washed three times with PBS containing 0.1% Tween 20 (PBS-T; both reagents from Sigma Laboratories, l'Isle d'Abeau Chesnes, France). The sera were diluted (1∶100) in PBS containing 0.1% Tween 20 and 5% nonfat dried milk (PBS-T-NDM) and added to each well (100 µl/well). Six negative and two positive reference sera were included in each plate as controls. The negative controls consisted of sera from patients with other febrile illnesses but negative for dengue infection. The plates were then incubated for 2 h at 37°C and washed three times with PBS-T. Bound IgG was detected by adding 100 µl of horseradish peroxidase-conjugated goat anti-human IgG (Jackson Immunoresearch Laboratories, West Grove, PA, USA) diluted 1∶2,000 in PBS-T-NDM to each well. The plates were incubated for 1 h at 37°C and washed with PBS-T. Bound antibodies were visualized by adding 100 µl of tetramethylbenzidine (Sigma Laboratories) to each well. After incubation for 15 min at room temperature, absorbance at 650 nm (A650) was read with a microplate reader, and the Δ_A650_ was calculated (Δ_A650_ = Δ_A650_ Den Ag−Δ_A650_ control Ag). For validation of the test, the Δ_A650_ of the positive control serum had to be ≥0.5 and the Δ_A650_ of the negative control serum had to be ≤0.2. The mean (±the standard deviation) Δ_A650_ values for the negative controls were then determined. A result was considered negative if Δ*_A_*_650_ was less than the mean value for the negative control plus two standard deviations, indeterminate when Δ*_A_*_650_ was between two and three standard deviations, and positive when Δ*_A_*_650_ values was more than three standard deviations greater than the mean of the negative control.

We studied a panel of 320 sera previously tested for NS1 antigen with the Platelia Dengue NS1 Ag kit [12]. Two hundred seventy-two DENV-infected sera were characterized as follows: (i) DENV-1 (n = 33), DENV-2 (n = 42), DENV-3 (n = 101) and DENV-4 (n = 46) acute sera (n = 222 in total), (ii) 50 non serotyped serum samples containing DENV IgM antibody detected during different dengue outbreaks from French Guiana (Table 1). We also included 48 additional acute-phase serum samples (days one to four) from patients presenting dengue-like syndrome (temperature of ≥38.5°C, arthralgia, headache and/or myalgia), for whom recent dengue infection was ruled out based on acute and convalescent sera. Of the 222 acute serum samples with dengue virus detected by RT-PCR and/or virus isolation, 194 (87.4%; 95% CI: 82.3% to 91.5%) were positive for the Platelia Dengue NS1 Ag test; four sera (1.8%) with equivocal results were considered negative based on further statistical analysis. We used these well-characterized sera to compare the performance of Dengue NS1 Ag STRIP and pan-E Dengue Early ELISA with the Platelia Dengue NS1 Ag test.

**Table 1:** Description of the panel with the DENV-infected serum samples (n = 272) used for evaluating the sensitivity of the Platelia Dengue NS1 Ag test, Dengue NS1 Ag STRIP and pan-E Dengue Early ELISA, according to the number of days after onset of fever.

Days after onset of fever	Acute phase (n = 222)				Early convalescence phase (n = 50)
	DENV-1	DENV-2	DENV-3	DENV-4	IgM
0	5	4	9	2	0
1	10	21	27	16	0
2	9	7	36	8	0
3	7	8	14	11	2
4	0	2	12	8	2
5	2	0	2	1	13
6	0	0	1	0	14
≥7	0	0	0	0	19
**Total**	**33**	**42**	**101**	**46**	**50**

Based on the WHO criteria for case definitions of dengue fever, DHF and DSS, all serum samples tested were collected from patients with clinical symptoms of classical dengue fever (fever, headache, myalgia, and arthralgia) with or without a rash or minor hemorrhagic manifestations. No patient presented severe clinical symptoms of DHF or DSS [18].

### New commercial tests based on dengue NS1 antigen detection

Dengue NS1 Ag STRIP (Bio-Rad Laboratories) is an immunochromatographic test (ICT) for the rapid detection of NS1 antigen. Briefly, one drop of migration buffer was added to 50 µL serum in a specimen tube and a strip was placed in the tube. The strip has two lines: a control line (C) (“biotin – gold colloidal particles coated with streptavidin” complex) and a test line (T) (“monoclonal anti-NS1 antibodies (mAb) – NS1 Ag – gold colloidal particles coated with anti-NS1 mAb” complex). The appearance of the T and C lines after a migration time of 15 minutes (min) indicates a positive result. The appearance of the C line alone indicates a negative result. If the C line is not present, the test is considered invalid and is repeated. It is recommended that strips giving ambiguous (faint color at the T line) or negative results are put back in the tube after the initial reading and left for a further 15 min for re-evaluation. We evaluated all samples at 15 min (ICT 15 min) and then at 30 min (ICT 30 min).

The pan-E Dengue Early ELISA (Panbio) is a two-step sandwich-format microplate enzyme-linked immunosorbent assay for the detection of NS1 antigen. The test uses polyclonal and monoclonal antibodies for capture and detection, respectively. Panel serum samples and control serum samples provided in the kit were diluted 1∶10 (135 µL of serum diluents and 15 µL of each serum sample and control sample). Diluted serum and control samples (100 µL) were placed in microplate wells coated with anti-NS1 polyclonal antibodies. The microplate was incubated for one hour at 37°C, and then washed six times. Horseradish peroxidase-conjugated anti-NS1 Mab (100 µL) was added to each well and the microplate was incubated for one hour at 37°C, then washed six times. Immune complexes were detected using a color development reaction (100 µL of tetramethylbenzidine added to each well). The plate was incubated for 10 minutes room temperature, and the enzymatic reaction was then stopped with phosphoric acid. Absorbance at 450/620 nm was determined within 30 minutes.

We used the average absorbance of triplicate readings from the calibrator, multiplied by the calibration factor (provided in the kit), to calculate the cut-off value (CO). An index value was calculated by dividing the sample absorbance by the CO. Panbio Units were calculated by multiplying the index value by ten. According to the manufacturer's recommendations, samples with index values less than 0.9 were considered negative (no detectable dengue NS1 Ag); those with index values between 0.9 and 1.1 were equivocal for the presence of dengue NS1 Ag, and samples with index values greater than 1.1 were considered positive (presence of detectable dengue NS1 Ag).

The technicians carrying out Dengue NS1 Ag STRIP and pan-E Dengue Early ELISA were blind to the DENV-infection status of the panel of serum samples. For each strip, two people read the results independently. When discordance was observed between Platelia and ICT and/or pan-E results, the discordant serum was retested with all three tests to avoid discrepancies due to the effects of serum sample storage.

Statistical analysis was performed with STATA version 9.0 (STATA Corporation, College Station, TX, USA).

## Results

The sensitivity of each of the three NS1 antigen detection tests with the 272 DENV-infected sera is detailed in Table 2. All sera testing positive for the presence of NS1 with the pan-E Dengue Early ELISA also tested positive with the Dengue NS1 Ag STRIP and Platelia Dengue NS1 Ag tests. Similarly, all sera testing positive for NS1 with the Dengue NS1 Ag STRIP also tested positive with the Platelia Dengue NS1 Ag test (Table 2). Using the Dengue NS1 Ag STRIP, 207 (76.1%; 95% CI: 70.6% to 81.0%) samples gave positive results at 15 minutes and 211 (77.6%; 95% CI: 72.1% to 82.4%) samples gave positive results at 30 minutes. One serum sample, collected on day four, gave ambiguous results at 15 minutes (faint Dengue NS1 Ag STRIP T line), and a positive result after 30 minutes (positive with the Platelia test, with a ratio greater than 5.0). A second serum sample, collected on day 0, was considered negative after 15 minutes and positive after 30 minutes (positive with the Platelia test, with a ratio of 3.9). With the pan-E Dengue Early ELISA, 150 serum samples (55.1%; 95% CI: 49.0% to 61.2%) tested positive. Nine other serum samples collected on day 1 (n = 1), day 2 (n = 2), day 3 (n = 2), day 4 (n = 2) and day 6 (n = 2) were considered equivocal with the pan-E Dengue Early ELISA, whereas these samples had ratios of 2.8 (n = 1) or greater than 5.0 (n = 8) with the Platelia test.

**Table 2:** NS1 Ag detection using Platelia Dengue NS1 Ag test, Dengue NS1 Ag STRIP and pan-E Dengue Early ELISA in patients with DENV-infection (n = 272) and among acute-phase serum samples from dengue-negative patients (n = 48).

Platelia Dengue NS1 Ag test		Dengue NS1 Ag STRIP 30 min.		Dengue NS1 Ag STRIP 15 min.		pan-E Dengue Early ELISA	
				Positive	207	Positive	149
		Positive	211			Negative	58
				Negative	4	Positive	0
Positive	224					Negative	4
				Positive	0	Positive	0
		Negative	13			Negative	0
				Negative	13	Positive	1
						Negative	12
Negative	48	Negative	48	Negative	48	Negative	48
**Se = 82.4% (224/272) [77.3–86.7]** a		**Se = 77.6% (211/272) [72.1–82.4]** a		**Se = 76.1% (207/272) [70.6–81.0]** a		**Se = 55.1% (150/272) [49.0–61.2]** a	
Negative	48	Negative	48	Negative	48	Negative	47
**Sp = 100% (48/48) [92.6–100]** b		**Sp = 100% (48/48) [92.6–100]** b		**Sp = 100% (48/48) [92.6–100]** b		**Sp = 97.9% (47/48) [88.9–99.9]** c	

a Se: Sensitivity (%), number of NS1 positive test among patients with confirmed DENV infection (n = 272) – [95% CI].

b Sp: Specificity (%), NS1 negative test among acute-phase serum samples from dengue-negative patients (n = 48) – [97,5% CI, one-sided test)].

c Sp: Specificity (%), NS1 negative test among acute-phase serum samples from dengue-negative patients (n = 48) – [95% CI].

Table 3 details the sensitivities of the three NS1 tests and of the MAC-ELISA according to the number of days after onset of fever. The sensitivity of ICT at 30 minutes was 94.2%, using the Platelia Dengue NS1 Ag test as a reference (100%), whereas that of the pan-E Dengue Early ELISA was 66.9%. The sensitivity of pan-E Dengue Early ELISA for DENV diagnosis between days 0 and 4 (60.0%) increased significantly, to 64.0%, when combined with IgM detection (McNemar's test: 9.0 p = 0.003) (Data not shown).

**Table 3:** Sensitivities of Platelia Dengue NS1 Ag test, Dengue NS1 Ag STRIP, pan-E Dengue Early ELISA, and of the MAC-ELISA according to the number of days after onset of fever in patients with DENV-infection (n = 272).

Day after onset of fever^a^	No. of sera tested	Platelia Dengue NS1 Ag test		Dengue NS1 Ag STRIP 15 min.		Dengue NS1 Ag STRIP 30 min.		pan-E Dengue Early ELISA		MAC-ELISA	
		No. of positive tests	Sensitivity (%) [95% CI]	No. of positive tests	Sensitivity (%) [95% CI]	No. of positive tests	Sensitivity (%) [95% CI]	No. of positive tests	Sensitivity (%) [95% CI]	No. of positive tests	Sensitivity (%) [95% CI]
0	20	19	95.0 [75.1–99.9]	16	80.0 [56.3–94.3]	17	85.0 [62.1–96.8]	13	65.0 [40.8–84.6]	1	5.0 [0–24.9]^b^
1	74	64	86.5 [76.5–93.3]	61	82.4 [71.8–90.3]	61	82.4 [71.8–90.3]	38	51.4 [39.4–63.1]	1	1.4 [0–7.3]^b^
2	60	56	93.3 [83.8–98.2]	51	85.0 [73.4–92.9]	51	85.0 [73.4–92.9]	40	66.7 [53.3–78.3]	3	5.0 [1.0–13.9]
3	42	36	85.7 [71.5–94.6]	35	83.3 [68.6–93.0]	35	83.3 [68.6–93.0]	27	64.3 [48.0–78.4]	7	16.7 [7.0–31.4]
4	24	18	75.0 [53.3–90.2	17	70.8 [48.9–87.4]	18	75.0 [53.3–90.2]	14	58.3 [36.6–77.9]	7	29.2 [12.6–51.1]
5	18	8	44.4 [21.5–69.2]	8	44.4 [21.5–69.2]	8	44.4 [21.5–69.2]	8	44.4 [21.5–69.2]	15	83.3 [58.6–96.4]
6	15	14	93.3 [68.1–99.8]	12	80.0 [51.9–95.7]	13	86.7 [59.5–98.3]	5	33.3 [11.8–61.6]	15	100 [78.2–100]^b^
≥7	19	9	47.4 [24.4–71.1]	7	36.8 [16.3–61.6]	8	42.1 [20.3–66.5]	5	26.3 [9.1–51.2]	19	100 [82.4–100]^b^
**Total**	**272**	**224**	**82.4 [77.3–86.7]**	**207**	**76.1 [70.6–81.0]**	**211**	**77.6 [72.1–82.4]**	**150**	**55.1 [49.0–61.2]**	**68**	**25.0 [20.0–30.6]**

a Onset of fever is defined as day 0 if blood samples were collected within the first 24 h after the onset of the disease.

b One-sided test, 97.5% confidence interval.

Table 4 compares the sensitivity of the Dengue NS1 Ag STRIP and pan-E Dengue Early ELISA tests with that of the Platelia Dengue NS1 Ag test, as a function of DENV serotype. No significant difference was observed between the four DENV serotypes for sensitivity of the Platelia test (χ^2^ = 0.49, *P* = 0.92), the ICT at 15 min (χ^2^ = 0.05, *P* = 0.99) or the ICT at 30 min (χ^2^ = 0.25, *P* = 0.97). However, a significantly lower sensitivity (21.7%; 95% CI: 10.9% to 36.4%) for DENV-4 serum samples was observed with the Panbio test (χ^2^ = 40.1, *P*<0.0001).

**Table 4:** Sensitivity of the Platelia Dengue NS1 Ag test, Dengue NS1 Ag STRIP and pan-E Dengue Early ELISA as a function of dengue virus serotype detected by RT-PCR and/or virus isolation (n = 222).

Serotype	No. of sera tested	Platelia Dengue NS1 Ag test		Dengue NS1 Ag STRIP 15 min.		Dengue NS1 Ag STRIP 30 min.		pan-E Dengue Early ELISA	
		No. of positive tests	Sensitivity % [95% CI]	No. of positive tests	Sensitivity % [95% CI]	No. of positive tests	Sensitivity % [95% CI]	No. of positive tests	Sensitivity % [95% CI]
DENV-1	33	30	90.9 [75.7–98.1]	27	81.8 [64.5–93.0]	27	81.8 [64.5–93.0]	28	84.8 [68.1–94.9]
DENV-2	42	36	85.7 [71.5–94.6]	34	81.0 [65.9–91.4]	34	81.0 [65.9–91.4]	30	71.4 [55.4–84.3]
DENV-3	101	88	87.1 [79.0–93.0]	82	81.2 [72.2–88.3]	83	82.2 [73.3–89.1]	66	65.3 [55.2–74.5]
DENV-4	46	40	87.0 [73.7–95.1]	38	82.6 [68.6–92.2]	39	84.8 [71.1–93.7]	10	21.7 [10.9–36.4]
**Total**	**222**	**194**	**87.4 [82.3–91.5]**	**181**	**81.5 [75.8–86.4]**	**183**	**82.4 [76.8–87.2]**	**134**	**60.4 [53.4–66.8]**

The sensitivity of Platelia Dengue NS1 Ag, ICT at 15 min and 30 min, and pan-E Dengue Early ELISA tests for acute sera with positive RT-PCR or viral culture results is presented in Table 5. No significant difference was observed between the sensitivity (Se) of ICT 15 min (Se = 81.8%; 180/220), ICT 30 min (Se = 82.7%; 182/220) and Platelia Dengue NS1 Ag (Se = 87.7%; 193/220) (χ^2^ = 3.3, *P* = 0.19) for acute serum samples collected between days 0 and 4 after fever onset. However, a significant difference in sensitivity was observed between pan-E Dengue Early ELISA (Se = 60.0%; 132/220) and Platelia Dengue NS1 Ag (χ^2^ = 43.8, *P*<0.0001), ICT 15 min (χ^2^ = 25.4, *P*<0.0001) and ICT 30 min (χ^2^ = 27.8, *P*<0.0001).

**Table 5:** Sensitivity of the Platelia Dengue NS1 Ag test, Dengue NS1 Ag STRIP and pan-E Dengue Early ELISA according to virus isolation or genome detection.

	No. of sera tested	Platelia Dengue NS1 Ag test		Dengue NS1 Ag STRIP 15 min.		Dengue NS1 Ag STRIP 30 min.		pan-E Dengue Early ELISA	
		No. of positive tests	Sensitivity % [95% CI]	No. of positive tests	Sensitivity % [95% CI]	No. of positive tests	Sensitivity % [95% CI]	No. of positive tests	Sensitivity % [95% CI]
Virus isolation	140	130	92.9 [87.3–96.5]	120	85.7 [78.8–91.1]	121	86.4 [79.6–91.6]	103	73.6 [65.5–80.7]
RT-PCR	118	98	83.1 [75.0–89.3]	94	79.7 [71.3–86.5]	95	80.5 [72.2–87.2]	62	52.5 [43.1–61.8]

**Figure 1:**
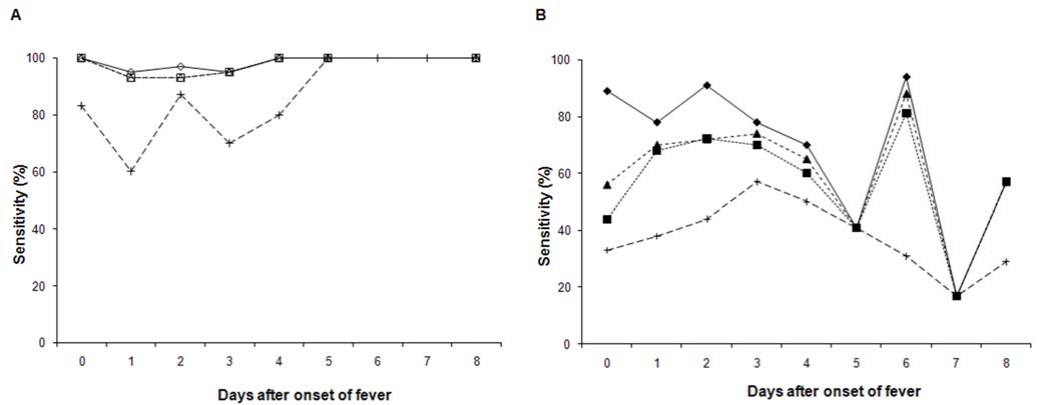
Sensitivity of the three tests for NS1 antigen detection, based on the presence or absence of IgG in the serum samples. A: Serum samples with no IgG: Platelia Dengue NS1 Ag test (—⋄—), Dengue NS1 Ag STRIP 15 min (- - -□- - -) and 30 min (– –▵– –), and pan-E Dengue Early ELISA (– –+– –). B: Serum samples positive for IgG: Platelia Dengue NS1 Ag test (—⧫—), Dengue NS1 Ag STRIP 15 min (- - -▪- - -) and 30 min, (– –▴– –) and pan-E Dengue Early ELISA (- - + - -).

There was a significant difference in the sensitivity of NS1 detection tests between serum samples with positive and negative results for DENV IgG detection (Platelia: χ^2^ = 24.5, *P*<0.0001; ICT 15 min. χ^2^ = 37.2, *P*<0.0001; ICT 30 min. χ^2^ = 33.3, *P*<0.0001; Pan-E: χ^2^ = 27.0, *P*<0.0001) (Table 6). This difference was observed regardless of the timing of serum sample collection (Figures 1A & 1B). Furthermore, we observed an increase in the sensitivity of the three NS1 detection tests between sera collected at day 0 and those collected on day 3, with the largest increase observed with the pan-E Dengue Early ELISA (Figure 1B).

**Table 6:** Sensitivity of the Platelia Dengue NS1 Ag test, Dengue NS1 Ag STRIP and pan-E Dengue Early ELISA according to IgG status (n = 272).

IgG detection	No. of sera tested	Platelia Dengue NS1 Ag test		Dengue NS1 Ag STRIP 15 min.		Dengue NS1 Ag STRIP 30 min.		Pan-E Dengue Early ELISA	
		No. of positive tests	Sensitivity % [95% CI]	No. of positive tests	Sensitivity % [95% CI]	No. of positive tests	Sensitivity % [95% CI]	No. of positive tests	Sensitivity % [95% CI]
Negative	109	105	96.3 [90.9–99.0]	104	95.4 [89.6–98.5]	104	95.4 [89.6–98.5]	81	74.3 [65.1–82.2]
Positive	163	119	73.0 [65.5–79.7]	103	63.2 [55.3–70.6]	107	65.6 [57.8–72.9]	69	42.3 [34.6–50.3]
**Total**	**272**	**224**	**82.4 [77.3–86.7]**	**207**	**76.1 [70.6–81.0]**	**211**	**77.6 [72.1–82.4]**	**150**	**55.1 [49.0–61.2]**

The specificity testing was conducted with the 48 acute-phase serum samples from dengue-negative patients. The Platelia Dengue NS1 Ag test, ICT 15 min and 30 min had a specificity of 100% (48/48; 97.5% CI: 92.6% to 100%, one-sided test), whereas the specificity of the pan-E Dengue Early ELISA was 97.9% (47/48; 95% CI: 88.9% to 99.9%). For one patient, we considered the positive Panbio test for the acute-phase serum sample to be a false positive result because virus isolation, genome and IgM detection tests were all negative. Moreover, for the 48 non-DENV-infected patients, all acute-phase and early convalescent sera were tested for IgM directed against two other flaviviruses (Saint-Louis encephalitis and yellow fever antigen, data not shown), and for the false-positive Panbio result, the paired sera were retested to confirm the absence of cross-reactivity with these two flaviviruses.

## Discussion

We compared three diagnostic tests for early detection of the NS1 antigen secreted in the plasma of patients with dengue infection during the acute phase of the disease. The Platelia Dengue NS1 Ag test has been already demonstrated to be effective for NS1 detection [12]–[13]. We show here a high rate of detection in samples from patients with the rapid immunochromatographic test, Dengue NS1 Ag STRIP — the first ICT test developed for NS1 detection — for all four DENV serotypes. This rapid diagnostic test (RDT) is convenient, easy to use, and the results are obtained in 15 minutes, or 30 minutes for ambiguous results. The RDT does not involve any specific laboratory equipment, except a microcentrifuge for serum separation. This test can detect NS1 Ag from serum samples, but not from whole blood. The development of an ICT for the NS1 detection in whole blood would allow rapid diagnosis of dengue in the field or during medical consultations in health care centers with minimal equipment. Moreover, an early ICT for NS1 could be performed in parallel with an RDT for malaria in tropical areas in which these two diseases are endemic.

The sensitivity of the pan-E Dengue Early ELISA was significantly lower than that of the Platelia Dengue NS1 Ag test or the Dengue NS1 Ag STRIP. This observation is consistent with a previous study performed in Southeast Asia with fewer serum samples (n = 92), in which the pan-E Dengue Early ELISA had a sensitivity of 63.2% (95% CI: 53.4% to 73.0%) for sera collected between days four and seven after fever onset [19]. We also show that this test differed significantly from the Platelia Dengue NS1 Ag and Dengue NS1 Ag STRIP tests for patients with the DENV-4 serotype and with acute serum samples collected between days 0 and four. Thus, the pan-E Dengue Early ELISA should not be used alone for acute diagnosis of DENV infection and should instead be combined with MAC-ELISA, as suggested by Blacksell *et al.* Furthermore, in practical terms, the Platelia Dengue NS1 Ag test, which has a one-step sandwich-format (one incubation period), is more convenient for technicians than the pan-E Dengue Early ELISA test, which has two steps (two incubation periods).

During the acute phase of the disease, the presence of DENV IgM antibody alone suggests primary infection, and the concomitant detection of DENV IgM and IgG antibodies is suggestive of secondary infection [18],[20]. The absence of DENV IgG antibody in serum samples collected between days 0 and eight makes it possible to classify the case as primary DENV infection. It has also been shown that, during the IgG antibody response, NS1-specific IgG antibodies start to appear on day 8 of primary infection and are still present during the first few days of DENV secondary infection [21]. As suggested by Table 6, the sensitivity of NS1 Ag detection with the three commercial tests would be expected to be higher during primary infection than during secondary infection, and we clearly showed greater sensitivity with the Platelia Dengue NS1 Ag test or the Dengue NS1 Ag STRIP than with the pan-E Dengue Early ELISA. This observation is consistent with recent findings with the Platelia Dengue NS1 Ag assay [13]. During these first few days of secondary infection, a substantial amount of NS1 protein may be bound to NS1-specific IgG antibodies from previous acute-phase infection and trapped within immune complexes [9],[22]. This difference in the kinetics of NS1-specific IgG antibodies between primary and secondary DENV infection is certainly similar to the kinetics observed for total DENV IgG antibodies. In addition, as observed in Table 3, the sensitivity of NS1 detection rapidly decreases on day five of secondary DENV infection, requiring NS1 detection between days 0 and four for an accurate diagnosis.

The accurate laboratory diagnosis of DENV infection is now possible with Dengue NS1 Ag detection tests, taking into account the timing of serum collection after the onset of fever. Previous studies have demonstrated a diagnostic strategy combining NS1 Ag detection in acute-phase sera and DENV IgM detection in early-convalescent-phase sera, providing a sensitivity of about 90% for dengue diagnosis [12],[23]. The development of a single immunochromatographic assay combining NS1 antigen and DENV IgM antibody detection would provide clinicians with a rapid test for dengue diagnosis sensitive during both the acute and early convalescence phases, between days 0 and 8 after the onset of fever.

NS1 detection has been shown to be suitable for early dengue diagnosis, whatever the DENV serotype responsible for dengue disease in Latin America and Southeast Asia, but only in two retrospective studies [12],[13]. Although these new diagnostic tools have been designed using two monoclonal antibodies (Platelia and ICT from Bio-Rad) or one monoclonal and one polyclonal antibody (pan-E from Panbio) for NS1 capture, and given the existence of different DENV genotypes within each serotype [24], it would be interesting to evaluate these two new diagnostic tools based on NS1 detection in prospective clinical studies in Asia and Latin America. It would also be useful to evaluate the performances of these kits taking into account different patient profiles such as ethnicity or disease severity. However, the severe fatal forms of the disease, such as DHF/DSS, are known to occur between four and six days after fever onset, whereas NS1 detection is optimized for all sera collected during the first five days of the disease.

We show that the sensitivity of NS1 detection with the Dengue NS1 Ag STRIP and Pan-E Dengue Early ELISA tests increased from day 0 to day 3 after fever onset, with the largest effect observed for serum samples without DENV IgG antibody. This phenomenon may be correlated with an increase in NS1 secretion over the first three days after fever onset [9]. This increase was not detectable with the Platelia Dengue NS1 Ag test, possibly due to the greater analytical sensitivity of the Platelia than of the Dengue NS1 Ag STRIP and pan-E Dengue Early ELISA tests.

We observed an increase in the sensitivity of the Platelia Dengue NS1 Ag and Dengue NS1 Ag STRIP tests on day 6 for serum samples positive for DENV IgG antibody, probably correlated with an increase in the amount of secreted NS1, but the number of serum samples was small (n = 15). Soluble NS1 protein binds to the human endothelium *in situ* [11] and accumulates in hepatocytes [25], possibly contributing to viral propagation. The release of soluble NS1 protein from the endothelium or from hepatocytes during DENV secondary infection may therefore play a role in the pathophysiology of the disease. Alternatively, the persistence of viral particles in capillary blood longer than in peripheral blood promotes the late release of soluble NS1 protein [26]. Further studies quantifying NS1 protein as a function of viral load in acute-phase and early convalescence-phase sera will provide further insight into these aspects.

In conclusion, this evaluation of two new commercial tests for dengue diagnosis, Dengue NS1 Ag STRIP and pan-E Dengue Early ELISA, and their comparison with the Platelia Dengue NS1 Ag test confirm that NS1 will be beneficial as a new marker in the diagnosis of acute DENV infection. The first rapid immunochromatographic test, Dengue NS1 Ag STRIP, which performs well, is suitable as a first-line test in the field, whereas pan-E Dengue Early ELISA should be used with DENV IgM antibody detection for the diagnosis of DENV infection.

## Supporting Information

Alternative Language Abstract S1 Translation of the Abstract into Portuguese by Pedro Fernando Vasconcelos, Instituto Evandro Chagas, Belém, Brazil
(0.05 MB PDF)

Alternative Language Abstract S2 Translation of the Abstract into Spanish by Samantha Brandler, Institut Pasteur, Paris, France
(0.05 MB PDF)

Alternative Language Abstract S3 Translation of the Abstract into French by Philippe Dussart
(0.05 MB PDF)
