# Supplementary material for: Evaluation of Two New Commercial Tests for the Diagnosis of Acute Dengue Virus Infection Using NS1 Antigen Detection in Human Serum
Source: PLoS Negl Trop Dis. 2008 Aug 20;2(8):e280. doi: 10.1371/journal.pntd.0000280 (PMC2500180; doi:10.1371/journal.pntd.0000280)
Supplement: Alternative Language Abstract S1 — Translation of the Abstract into Portuguese by Pedro Fernando Vasconcelos, Instituto Evandro Chagas, Belém, Brazil (0.05 MB PDF) [file pntd.0000280.s001.pdf]

Translation of the abstract in Portuguese by Pedro Fernando Vasconcelos, Instituto Evandro Chagas, Belém, Brazil


## RESUMO

**Introdução:** Nós comparamos o desempenho de dois novos testes comerciais disponíveis para a detecção da proteína NS1 durante a fase clínica da infecção causada pelo vírus dengue (VDEN) com o ELISA em uma etapa, Platelia ™ Dengue NS1 Ag Test (Bio-Rad): um em formato de teste imunocromatográfico que permite a detecção rápida do antígeno NS1, o Ag STRIP (Bio-Rad Laboratories-Marnes La Coquette, France), e outro em formato de ELISA sanduíche em duas etapas, pan-E Dengue Early ELISA (Panbio, Brisbane, Austrália).
**Metodologia:** O total de 272 amostras de soros de pacientes com dengue foi utilizado para a avaliação dos kits. Destas, 222 amostras foram obtidas de pacientes com infecção aguda causada por um dos quatro sorotipos do VDEN, confirmadas por RT-PCR e/ou isolamento viral. Quarenta e oito soros coletados de pacientes não infectados pelo VDEN foram incluídos no estudo.
**Resultados:** A sensibilidade do kit Platelia ™ Dengue NS1 Ag Test em amostras de soros agudos (n=222) foi de 87,4% ([IC] intervalo de confiabilidade de 95%: 82,3% a 91,5%) ; para o Dengue NS1 STRIP, após 15 e 30 minutos, a sensibilidade foi de 81,5% (IC 95%: 75,8% a 86,4%) e 82,4% (IC 95%: 76,8% a 87,2%), respectivamente. Ambos os testes mostraram especificidade de 100% (IC 97,5%: 92,6% a 100,0%). O kit pan-E Dengue Early ELISA apresentou sensibilidade de 60,4% (IC 95%: 53,4% a 66,8%) e especificidade de 97,9% (IC 95%: 88,9% a 99,9%).
**Conclusão:** Nossos resultados sugerem o uso de métodos que tenham como base a detecção de antígeno NS1 para o diagnóstico de infecções agudas causadas pelo VDEN. O teste imunocromatográfico, Dengue NS1 Ag STRIP, o primeiro teste efetivamente rápido para o diagnóstico da dengue, foi altamente sensível e específico, e poderia, portanto ser utilizado como teste de primeira linha em análises durante epidemias. O kit pan-E Dengue Early ELISA foi menos sensível em comparação ao teste Platelia; este ELISA em duas etapas poderia ser associado à detecção de anticorpos IgM para o diagnóstico da dengue em sua fase aguda.
