# Supplementary material for: Evaluation of Two New Commercial Tests for the Diagnosis of Acute Dengue Virus Infection Using NS1 Antigen Detection in Human Serum
Source: PLoS Negl Trop Dis. 2008 Aug 20;2(8):e280. doi: 10.1371/journal.pntd.0000280 (PMC2500180; doi:10.1371/journal.pntd.0000280)
Supplement: Alternative Language Abstract S2 — Translation of the Abstract into Spanish by Samantha Brandler, Institut Pasteur, Paris, France (0.05 MB PDF) [file pntd.0000280.s002.pdf]

Translation of the abstract in Spanish by Samantha Brandler, Institut Pasteur, Paris, France

RESUMEN

Antecedentes: Se compararon los resultados de dos nuevos métodos comerciales disponibles para la detección de la proteína del dengue NS1 durante la fase aguda de la infección: el método inmunocromatográfico (ICT) de diagnostico rápido “Dengue NS1 Ag STRIP” (Bio-Rad Laboratories - Marnes La Coquette, Francia), y el método ELISA por captura del antígeno NS1 del dengue “pan-E Dengue Early ELISA” (Panbio - Brisbane, Australia). Ambas técnicas fueron comparadas con el método ELISA “Platelia™ Dengue NS1 Ag test” (Bio-Rad) desarrollado en una sola etapa.

Métodos: Se analizaron 272 sueros provenientes de pacientes con diagnóstico de dengue. De éstos, 222 sueros de pacientes con infección aguda por uno de los cuatro serotipos del dengue, detectado por RT-PCR y/o aislamiento del virus. Cuarenta y ocho muestras de suero de pacientes en fase aguda no infectados con el virus del dengue fueron incluidos en este estudio.

Resultados: La sensibilidad del método “Platelia Dengue NS1 Ag” en las muestras de suero en fase aguda (n=222) fue del 87.4% (intervalo de confianza del 95%: 82.3% a 91.5%); la del método “Dengue NS1 Ag STRIP” después de 15 y 30 minutos fue del 81.5% (IC del 95%: 75.8% a 86.4%) y 82.4% (IC del 95%: 76.8% a 87.2%), respectivamente. Ambos métodos demostraron una especificidad del 100% (97.5% IC: 92.6% a 100.0%). El test “pan-E Dengue Early ELISA” demostró una sensibilidad del 60.4% (IC del 95%: 53.4% a 66.8%) y una especificidad del 97.9% (IC del 95%: 88.9% a 99.9%).

Conclusión: Nuestros resultados apoyan el uso de los métodos basados en la detección del antígeno NS1 para el diagnostico de la infección por virus del dengue durante la fase aguda. El método inmunocromatográfico (ICT) de diagnostico rápido “Dengue NS1 Ag STRIP” – el primer método de diagnóstico rápido de infección por dengue – demostró una alta sensibilidad y especificidad, y podría ser recomendado para la detección de casos de dengue en terreno. El método “pan-E Dengue Early ELISA” desarrollado en dos etapas demostró una sensibilidad inferior a la del método “Platelia Dengue NS1 Ag”: este método ELISA en dos etapas se debe combinar con la detección de anticuerpos IgM anti dengue para el diagnostico de la dengue.
