# Supplementary material for: Evaluation of Two New Commercial Tests for the Diagnosis of Acute Dengue Virus Infection Using NS1 Antigen Detection in Human Serum
Source: PLoS Negl Trop Dis. 2008 Aug 20;2(8):e280. doi: 10.1371/journal.pntd.0000280 (PMC2500180; doi:10.1371/journal.pntd.0000280)
Supplement: Alternative Language Abstract S3 — Translation of the Abstract into French by Philippe Dussart (0.05 MB PDF) [file pntd.0000280.s003.pdf]

Translation of the abstract into French by Philippe Dussart

RÉSUMÉ

Contexte: Nous avons comparé les performances de deux nouveaux kits commerciaux permettant la détection de la protéine NS1, protéine non structurale sécrétée par le virus de la dengue pendant la phase aiguë de la maladie : le premier kit "Dengue NS1 Ag STRIP" (Bio-Rad Laboratories - Marnes La Coquette, France) est un test de diagnostic rapide de type immunochromatographique (ICT), le second kit "pan-E Dengue Early ELISA" (Panbio - Brisbane, Australie) est un test ELISA développé en deux étapes pour la capture de l'antigène NS1. Les performances de ces deux nouveaux outils diagnostiques ont été comparées à celles du test ELISA "Platelia™ Dengue NS1 Ag test" (Bio-Rad), test ELISA développé en une seule étape.
Méthode: Nous avons testés 272 sérums de patients infectés par le virus de la dengue ; pour 222 sérums l'un des quatre sérotypes de dengue était identifié (sérums précoces). L'infection a été confirmée chez ces patients par RT-PCR et/ou culture cellulaire. Quarante-huit sérums précoces de patient non infectés par le virus de la dengue ont également été inclus dans cette étude.
Résultats: La sensibilité du test "Platelia Dengue NS1 Ag" sur les sérums précoces était de 87,4% (IC 95% : 82,3% à 91,5%); la sensibilité du kit "Dengue NS1 Ag STRIP" après une lecture de la bandelette (Strip) à 15 et 30 minutes était respectivement égale à 81,5% (IC 95% : 75,8% à 86,4%) et 82,4% (IC 95% : 76,8% à 87,2%). Ces deux tests ont montré une spécificité de 100% (IC 97,5% : 92,6% à 100,0%). Le test "pan-E Dengue Early ELISA" a montré une sensibilité de 60,4% et une spécificité de 97,9% (IC 95% : 88,9% à 99,9%).
Conclusion: Notre étude confirme l'intérêt de l'utilisation de tests basés sur la détection de la protéine NS1 pour le diagnostic d'infection aiguë par le virus de la dengue. Le test immunochromatographique "Dengue NS1 Ag STRIP" – premier test rapide développé pour le diagnostic de la dengue – a montré de très bonnes sensibilité et spécificité et pourrait être recommandé sur le terrain comme test de première intention. Le test "pan-E Dengue Early ELISA" développé en deux étapes a montré une sensibilité inférieure à celle du "Platelia Dengue NS1 Ag". Pour palier son manque de sensibilité, le test "pan-E Dengue Early ELISA" devrait être associé à un test sérologique permettant la détection des IgM anti-dengue.
